# Supplementary material for: Competition and stereotypic behavior in Thoroughbred horses: The value of saliva as a diagnostic marker of stress
Source: PLoS One. 2024 Oct 7;19(10):e0311697. doi: 10.1371/journal.pone.0311697 (PMC11458048; doi:10.1371/journal.pone.0311697)
Supplement: Attachment — Submitted filename: PONE-D-24-20481_reviewer comments.pdf [file pone.0311697.s002.pdf]

PLOS ONE

Agonism and stereotypes in thoroughbreds horses: the value of saliva as diagnostic marker of stress

--Manuscript Draft--

Manuscript Number:	PONE-D-24-20481
Article Type:	Research Article
Full Title:	Agonism and stereotypes in thoroughbreds horses: the value of saliva as diagnostic marker of stress
Short Title:	Salivary analysis in thoroughbreds horses
Corresponding Author:	Andrea Marchegiani, D.V.M., Ph.D.<br>Camerino University: Universita degli Studi di Camerino<br>Matelica, MC ITALY
Keywords:	horse; stereotypy; behavior; stress; saliva; cortisol; butyrylcholinesterase; salivary alpha amylase
Abstract:	Many horses exhibit stereotypies mainly when living in human controlled environments and natural needs in terms of feeding, drinking, moving, and socializing cannot be satisfied. In human medicine, obsessive compulsive disorder and other severe psychiatric disturbances are associated with stereotypic behaviors and salivary evaluation is considered a reliable tool for diagnosis of stress, anxiety, depression, and other mental disorders being saliva easy to collect and noninvasive. In this study, we hypothesized that cortisol, alpha amylase, and butyrylcholinesterase levels in saliva may be influenced by stereotypic behavior in horses. Saliva was obtained from ten healthy thoroughbreds horses stabled in individual boxes and used for agonistic competition; 11 healthy thoroughbreds horses stabled in individual boxes and used for agonistic competition showing stereotypic behaviors, and 5 thoroughbreds horses stabled in individual boxes, performing light physical activity for leisure purposes. Cortisol was found to be higher in agonistic horses and salivary alpha amylase was significantly higher in leisure horses when compared to other ones, while butyrylcholinesterase did not change between groups. These results may represent the basis for further evaluation to elucidate how agonistic and stereotyped horses cope with stressful situations and to monitor over time possible interventions established in attempt to improve welfare and health.
Order of Authors:	Marilena Bazzano
	Andrea Marchegiani, D.V.M., Ph.D.
	Francesca La Gualana
	Begi Petriti
	Marina Petrucelli
	Lucrezia Accorroni
	Fulvio Laus
Additional Information:	
**Question**	**Response**
**Financial Disclosure**<br><br>Enter a financial disclosure statement that describes the sources of funding for the work included in this submission. Review the submission guidelines for detailed requirements. View published research articles from *PLOS ONE* for specific	The author(s) received no specific funding for this work.

Powered by Editorial Manager® and ProduXion Manager® from Aries Systems Corporation

examples.<br><br>This statement is required for submission and **will appear in the published article** if the submission is accepted. Please make sure it is accurate.<br><br>**Funded studies**<br>Enter a statement with the following details:<br>• Initials of the authors who received each award<br>• Grant numbers awarded to each author<br>• The full name of each funder<br>• URL of each funder website<br>• Did the sponsors or funders play any role in the study design, data collection and analysis, decision to publish, or preparation of the manuscript?<br><br>Did you receive funding for this work?	
**Competing Interests**<br><br>Use the instructions below to enter a competing interest statement for this submission. On behalf of all authors, disclose any competing interests that could be perceived to bias this work—acknowledging all financial support and any other relevant financial or non-financial competing interests.<br><br>This statement is required for submission and **will appear in the published article** if the submission is accepted. Please make sure it is accurate and that any funding sources listed in your Funding Information later in the submission form are also declared in your Financial Disclosure statement.<br><br>View published research articles from *PLOS ONE* for specific examples.	The authors have declared that no competing interests exist.

**NO authors have competing interests**<br><br>Enter: *The authors have declared that no competing interests exist.*<br><br>**Authors with competing interests**<br><br>Enter competing interest details beginning with this statement:<br><br>*I have read the journal's policy and the authors of this manuscript have the following competing interests: [insert competing interests here]*<br><br>* typeset	
**Ethics Statement**<br><br>Enter an ethics statement for this submission. This statement is required if the study involved:<br><br>• Human participants<br>• Human specimens or tissue<br>• Vertebrate animals or cephalopods<br>• Vertebrate embryos or tissues<br>• Field research<br><br>Write "N/A" if the submission does not require an ethics statement.<br><br>General guidance is provided below. Consult the submission guidelines for detailed instructions. **Make sure that all information entered here is included in the Methods section of the manuscript.**	This study was carried out in strict accordance with the recommendations in the Guide for the Care and Use of Laboratory Animals of the National Institutes of Health. The protocol was approved by the Committee on the Ethics of Animal Experiments of the University of Camerino (Protocol Number: 10/2023) and were in accordance with the standards recommended by the EU Directive 2010/63/EU for experiments on animals.

Powered by Editorial Manager® and ProduXion Manager® from Aries Systems Corporation

**Format for specific study types**<br><br>**Human Subject Research (involving human participants and/or tissue)**<br>• Give the name of the institutional review board or ethics committee that approved the study<br>• Include the approval number and/or a statement indicating approval of this research<br>• Indicate the form of consent obtained (written/oral) or the reason that consent was not obtained (e.g. the data were analyzed anonymously)<br><br>**Animal Research (involving vertebrate animals, embryos or tissues)**<br>• Provide the name of the Institutional Animal Care and Use Committee (IACUC) or other relevant ethics board that reviewed the study protocol, and indicate whether they approved this research or granted a formal waiver of ethical approval<br>• Include an approval number if one was obtained<br>• If the study involved *non-human primates*, add *additional details* about animal welfare and steps taken to ameliorate suffering<br>• If anesthesia, euthanasia, or any kind of animal sacrifice is part of the study, include briefly which substances and/or methods were applied<br><br>**Field Research**<br><br>Include the following details if this study involves the collection of plant, animal, or other materials from a natural setting:<br>• Field permit number<br>• Name of the institution or relevant body that granted permission	
**Data Availability**<br><br>Authors are required to make all data underlying the findings described fully available, without restriction, and from the time of publication. PLOS allows rare exceptions to address legal and ethical concerns. See the PLOS Data Policy and FAQ for detailed information.	Yes - all data are fully available without restriction

Powered by Editorial Manager® and ProduXion Manager® from Aries Systems Corporation

A Data Availability Statement describing where the data can be found is required at submission. Your answers to this question constitute the Data Availability Statement and **will be published in the article**, if accepted.<br><br>**Important:** Stating 'data available on request from the author' is not sufficient. If your data are only available upon request, select 'No' for the first question and explain your exceptional situation in the text box.<br><br>Do the authors confirm that all data underlying the findings described in their manuscript are fully available without restriction?	
**Describe where the data may be found in full sentences. If you are copying our sample text, replace any instances of XXX with the appropriate details.**<br><br>• If the data are **held or will be held in a public repository**, include URLs, accession numbers or DOIs. If this information will only be available after acceptance, indicate this by ticking the box below. For example: *All XXX files are available from the XXX database (accession number(s) XXX, XXX.*).<br>• If the data are all contained **within the manuscript and/or Supporting Information files**, enter the following: *All relevant data are within the manuscript and its Supporting Information files.*<br>• If neither of these applies but you are able to provide **details of access elsewhere**, with or without limitations, please do so. For example:<br><br>*Data cannot be shared publicly because of [XXX]. Data are available from the XXX Institutional Data Access / Ethics Committee (contact via XXX) for researchers who meet the criteria for access to confidential data.*<br><br>*The data underlying the results presented in the study are available from (include the name of the third party*	The data underlying the results presented in the study are available from https://docenti.unicam.it/pdett.aspx?ids=N&tv=d&UteId=1120&ru=RD

and contact information or URL).<br>• This text is appropriate if the data are owned by a third party and authors do not have permission to share the data.<br><br>* typeset	
Additional data availability information:	Tick here if your circumstances are not covered by the questions above and you need the journal’s help to make your data available.; Tick here if the URLs/accession numbers/DOIs will be available only after acceptance of the manuscript for publication so that we can ensure their inclusion before publication.

Manuscript Click here to access/download;Manuscript;Manuscript.docx

Agonism and stereotypes in thoroughbreds horses: the value of saliva as diagnostic marker of stress

Marilena Bazzano[1], Andrea Marchegiani[1*], Francesca La Gualana [2], Begi Petriti[2], Marina Petrucelli[1], Lucrezia Accorroni[1], Fulvio Laus[1]

[1]School of Biosciences and Veterinary Medicine, University of Camerino, Via Circonvallazione, 93/95, 62024 Matelica, Italy

[2]Department of Translational and Precision Medicine, Sapienza, University of Rome (RM) -Italy

Author contributions

Conceptualization: MB; Data curation: MB, AM; Formal analysis: MB, AM, FLG, BP, MP, LA, FL; Investigation: MB, AM, FLG, BP; Methodology: FLG, BP, LA; Writing—original draft: MB, AM, FLG, BP, MP; Writing—review & editing: MB, AM, FLG, BP, FL.

Abstract

Many horses exhibit stereotypies mainly when living in human controlled environments and natural needs in terms of feeding, drinking, moving, and socializing cannot be satisfied. In human medicine, obsessive compulsive disorder and other severe psychiatric disturbances are associated with stereotypic behaviors and salivary evaluation is considered a reliable tool for diagnosis of stress, anxiety, depression, and other mental disorders being saliva easy to collect and noninvasive. In this study, we hypothesized that cortisol, alpha amylase, and butyrylcholinesterase levels in saliva may be influenced by stereotypic behavior in horses. Saliva was obtained from ten healthy thoroughbreds horses stabled in individual boxes and used for agonistic competition; 11 healthy thoroughbreds horses stabled in individual boxes and used for agonistic competition showing stereotypic behaviors, and 5 thoroughbreds horses stabled in individual boxes, performing light physical activity for leisure purposes. Cortisol was found to be higher in agonistic horses and salivary alpha amylase was significantly higher in leisure horses when compared to other ones, while butyrylcholinesterase did not change between groups. These results may represent the basis for further evaluation to elucidate

how agonistic and stereotyped horses cope with stressful situations and to monitor over time possible interventions established in attempt to improve welfare and health.

Keywords: horse, stereotypy, behavior, stress, saliva, cortisol, butyrylcholinesterase, salivary alpha amylase.

Introduction

Stereotypies are defined as compulsive and monotonous behavioral patterns performed repetitively without a defined target [1]. These abnormal communicative responses have been described in wild animals kept in captivity, in domesticated animals, and in people affected by mental disorders [2]. In equines, stereotypies have not been observed in wild horses living in their natural habitat, but they can be easily exhibited by domesticated horses living in human controlled environments when natural needs, in terms of feeding, drinking, moving, or socializing, cannot be satisfied [3,4]. Just as for human beings, also in animals' physiology stress is considered a major event able to strongly influence animal homeostasis, psychology, and cognition [5].

In human medicine, stereotyped behaviors have been associated with neurological disorders or psychiatric disturbances as schizophrenia, autism, obsessive compulsive disorder, and Tourette's syndrome. Different types of stereotypes have been described within equids in response to stressor stimuli [6–9]. Hausberger et al (2007) observed that stereotyped horses are less successful in learning ability than healthy horses and require more time to perform specific tasks[10]. However, more recent studies challenged this assumption, founding no difference between healthy and stereotyped horses if they are not prevented to perform the stereotyped behavior [11,12]. According to this knowledge, preventing a horse from exhibiting stereotypies by using cribbing collars, drugs, or surgery – unless life-threatening – may impede the horse's ability to cope with stressful situations [13].

Irrespective of the type of horse stereotypy, different biological parameters can be measured to determine the possible link between compulsive behavioral patterns and stress. Cortisol has been the most studied stress hormone[14], indispensable for animal survival and adaptation strategy (i.e. is the main actor of "fly or fight" reaction). When chronically secreted in elevated concentrations, cortisol can produce detrimental effects, negatively impacting on heart and respiratory rates, hydration status, body temperature, and lactic acid production. Several studies pointed the positive correlation between cortisol concentrations and stereotypical behavior occurrence [15–17].

Alongside with cortisol, in recent years other parameters are being considered as markers of stress in horses, including salivary alpha-amylase (sAA) and salivary butyrylcholinesterase (BChol) [18]. These metabolites have been also investigated in human saliva, with interesting associations with stress and mental disorders such as anxiety, depression and attention deficit hyperactivity [19–23] Recently, saliva is gaining increasing popularity as biological fluid for diagnostic investigations, in both human and veterinary medicine, due to non-invasive procedure that allows minimizing sampling stress especially for pediatric and animal patients [21,24,25].

In this study we analyzed salivary cortisol, alpha amylase, and butyrylcholinesterase concentrations in three types of sport horses, namely healthy agonistic, stereotyped agonistic and healthy non-agonistic ones, by supposing that agonistic activity and/or stereotyped behaviors could be correlated with salivary stress markers.

Materials and methods

Animals

This study was carried out in strict accordance with the recommendations in the Guide for the Care and Use of Laboratory Animals of the National Institutes of Health. The protocol was approved by the Committee on the Ethics of Animal Experiments of the University of Camerino (Protocol Number: 10/2023) and were in accordance with the standards recommended by the EU Directive 2010/63/EU for experiments on animals.

A total of 26 thoroughbreds were included in the study with informed owners' consent. Group A (agonistic) included 10 clinically healthy horses (4 males, 6 females, mean age 2.7±1.1 years) stabled in individual boxes, performing regular training for agonistic competition consisting of one-hour daily session (warm-up - trot and gallop sections - cool down). Group S (stereotyped) included 11 horses (5 males, 6 females; mean age 3.4±1.3 years), stabled in individual boxes, performing the same training of group A, showing no clinical sign of disease but stereotypic behaviors (crib-biting n. 7, weaving n. 2, box walking n. 2) at least since a year prior. Group L (non-agonistic) included 5 healthy horses (3 females, 2 geldings; mean age 7.2±3.5 years) stabled in individual boxes, performing equestrian leisure (principally hacking and trekking at weekends) but not involved in agonistic performance.

Sampling

Saliva samples were collected in November 2022, in the morning (07:00 a.m. - 08:00 a.m.), at stall rest, before animal feeding, using cotton swabs (Salivette® Sarstedt AG & Co., Nümbrecht, Germany) inserted into a customized mouthpiece that horses chewed for 5 minutes (figure 1).

Figure 1. Sampling tool used to collect saliva.

Horses were allowed to familiarize with the sampling tool few days prior the beginning of samplings. Samples from agonistic horses were obtained three to four days after competition. After each collection, cotton swabs were removed from the mouthpiece by means of surgical clamps, cooled immediately and delivered to the laboratory within 2 hours.

Laboratory analysis

Saliva samples were obtained by centrifugation of Salivette® tubes (20 min at 3000*g*) (Universal 32, Hettich Zentrifugen, Tuttlingen, Germany) and 0.5 mL aliquots of saliva were stored at −20 °C until analysis. At the day of the analysis saliva samples were quickly thawed at 37°C and centrifuged (10 min at 1000g) to eliminate debris residues. Surnatants were carefully harvested. Salivary cortisol, butyrylcholinesterase, and amylase levels were measured using Horse Cholinesterase ELISA Kit, Horse Cortisol ELISA Kit, Horse Amylase ELISA kit (all from Bioassay Technology Laboratory), respectively, according to the Manufacturer’s instructions. The absorbance was determined with Thermo Scientific Multiskan Sky amounted to 450 nm. GraphPad Prism version 8.2.1 for macOS (GraphPad Software, La Jolla California USA) was used to create a standard curve and to calculate the concentration of each analyte.

Statistical analysis

GraphPad Prism version 8.2.1 for macOS (GraphPad Software, La Jolla California USA) was used to perform the statistical analyses. First, data were checked using Shapiro-Wilk test for normality, showing sAA, salivary butyrylcholinesterase, and cortisol a normal distribution. Then, ordinary one-way ANOVA was performed to assess if concentrations of salivary biomarkers were statistically different between non-agonistic, agonistic, and stereotyped horses. After, Dunnett's multiple comparisons test was applied to compare data from agonistic and stereotyped horses with non-agonistic ones. Values of $p<0.05$ were considered significant.

Results

The mouthpiece used for sampling was well tolerated by all horses (no signs of discomfort or stress were displayed during collection) as well as did not cause neither a worsening of the pre-existing stereotypes nor the display of new ones in stereotyped horses, allowing an easy-going collection in all animals.

SAA activity, salivary butyrylcholinesterase (BChol) and cortisol concentrations in the three different groups are reported in Table 1.

Table 1. Descriptive statistics for salivary biomarkers

	**leisure**				**athletic**				**stereotypes**			
	mean value±sd	*min.*	*max.*	*range*	*mean value±sd*	*min.*	*max.*	*range*	*mean value±sd*	*min.*	*max.*	*range*
**sAA**	52.22 ± 12.44	34.40	66.70	32.30	38.48 ± 7.82	24.70	54.90	30.20	38.96 ± 10.71	20.70	52.20	31.50
**BChol**	6.52 ± 2.25	4.20	9.90	5.70	8.54 ± 2.35	5.10	12.80	7.70	6.08 ± 1.65	3.80	9.60	5.80
**Cortisol**	9.30 ± 8.40	0.00	19.40	19.40	30.10 ± 9.26	15.10	43.40	28.30	19.29 ± 11.39	0.90	43.80	42.90

Data are expressed in U/L, mU/mL, and ng/mL for salivary alpha-amylase (sAA), butyrylcholinesterase (BChol), and cortisol, respectively.

min: minimum concentration; *max*: maximum concentration

Statistical analysis showed a significant difference for sAA concentrations among groups ($p=0.04$). When applying Dunnett's comparisons test (figure 2), sAA in non-agonistic horses was significantly different with respect to agonistic and stereotyped ones ($p=0.03$ and $p=0.04$, respectively).

BChol was significantly different among groups ($p=0.03$), but not for multiple comparisons test.

Cortisol was significantly lower in non-agonistic horses than agonistic and stereotyped ones ($p=0.003$), whereas agonistic horses showed a significant higher level of cortisol when compared to non-agonistic horses ($p=0.002$ for Dunnett's test).

Figure 2. SAA activity, BChol, and cortisol measured in non-agonistic (L), agonistic (A), and stereotyped horses (S). Asterisk indicates statistically significant differences for Dunnett's test (* $p<0.05$, ** $p=0.001$) between groups.

Discussion

Saliva is considered a valuable biological fluid for stress assessment in horses as its sampling is usually non-invasive and well tolerated by animals [19,24]. In addition, it has the further advantage to accurately reflect the free and active steroid hormones levels of serum [26] without suffering from abrupt fluctuation due to stressful event, including the procedure of blood sampling for some horses[14]. Contreras-Aguilar and colleagues assessed the influence of the presence of food in mouth on salivary metabolites evaluation [27]. We sampled saliva in the morning before the horses were fed and so it is expected to have avoided such unfavorable

Cortisol has been the first hormone linked to stress and the association between stereotypies and its levels in horses is still debated [15]. Several studies on this topic came to conflicting results; few authors supported the existence of correlations between stress hormones and compulsive behavioral pattern [28,29] whereas others found no significant correlation [7,16,17].

In our study, we found significant statistical differences in salivary cortisol concentrations between non-agonistic and agonistic horses, with higher salivary cortisol in subjects involved in agonistic competitions. A possible explanation of the higher basal levels of salivary cortisol could be consequence of the racing activity regularly performed by these horses, as the management and the type of training was homogeneous among horses. However, Massányi and colleagues found no differences in salivary cortisol measured in horse experimenting different kind of stress linked to

sport activity (training, competition, shoeing, etc.) except for transportation [30]. In the present study, healthy agonistic horses showed higher salivary cortisol levels also in comparison to stereotyped horses, supporting the hypothesis that stereotypies have an anti-stress function as demonstrated in people [31] and in horses [13]. McBride and Cuddeford reported that plasma cortisol levels were higher immediately prior to exhibiting a stereotyped behavior (crib-biting bout), followed by a significant reduction after (post-crib-biting) [13]. Following an ACTH challenge test to experimentally induce a stress reaction, Briefer Freymond and colleagues found that crib-biters had significantly higher salivary cortisol than controls (non-stereotyped horse), with the highest levels observed in crib-biters that did not show stereotypy during the test [32,33]. Our results are in line with scientific literature evidence supporting the hypothesis that stereotyped behaviors represent coping strategies that help the affected animal to reduce stress levels, resulting in lowering of salivary stress hormones concentrations [13].

Besides cortisol, other salivary markers including alpha-amylase (sAA) and butyrylcholinesterase have been proposed to discriminate between stress and anxiety in people and animals, as well as to diagnose depressive disorders in humans.[24,34] In the present study, the highest sAA levels were recorded in non-agonistic horses, with agonistic and stereotyped horses showing lower values. SAA levels has been shown to increase following acute stressors, as abdominal disease [35] and acute fear-inducing stressors [18,36]. Recent research by Kusmawan and colleagues found that occupational fatigue and sleep quality are significantly correlated with sAA concentration in humans, proposing sAA as a potential noninvasive biological marker of sleep quality [37]. Agonistic and stereotyped horses included in the present study were adapted to their condition and the desensitization to proper stressors may explain the lower sAA values than non-agonistic horses. BChol is related to an increasing of sympathetic tone in horses and can be potentially used to assess exercise-related stress after a physical effort in endurance horses [38] or as marker of acute stress [18]. The role of BChol in saliva is unknown and no differences were found in the present study among agonistic, non-agonistic and stereotyped horses, probably due to the lack of acute stressor events or as a part of copying strategy. In our previous study on free ranging and paddock-stabled horses, BChol salivary concentrations were significantly higher in saliva samples collected in the wild compared to samples collected on the same mares restrained in paddocks[39].

The results of sAA and BChol may have been suffered from the low number of horses included in the study, for which environmental stressor stimuli may have cause an acute source of stress. Further investigation including a larger number of non-agonistic horses may help to elucidate theses aspects.

The present study has other limitations. Hair cortisol concentration has not been evaluated and it would have been beneficial to confirm the laboratory results.

Non agonistic and stereotyped horses included in the present study, which have been expected to experience more stress than agonistic ones, may have already put in place the adaptive strategy called “coping” to face a new, potentially stressful situation through behavioral and physiological modifications. Since it is not possible to diagnose it, it cannot be excluded that the results of the present study have been influenced by copying strategy.

Conclusions

When a stressful condition occurs, horses need to cope with the new situation using both behavioral and physiological struggles. Some of these parameters can be directly evaluated in saliva, providing new and dependable insights into the complex mechanism of stress and its regulation. Different physiological animals-based parameters can be evaluated and interpreted in light of behavioral responses, requiring further studies.

In this context, scientific research for understanding the individual adaptive capacity to stressful events in agonistic, non-agonistic, and stereotyped horses represent a milestone toward the welfare assessment of animals.

References

1. Radkowska I, Godyń D, Fic K. Stereotypic behaviour in cattle, pigs and horses-a review. Genetics and Animal Biotechnology of the Polish Academy of Sciences. 2020.
2. Mason GJ. Stereotypic Animal Behaviour: Fundamentals and Applications to Welfare and beyond. Stereotypic Animal Behaviour: Fundamentals and Applications to Welfare. 2008; 367. Available: https://www.nhbs.com/stereotypic-animal-behaviour-book
3. Hanis F, Chung ELT, Kamalludin MH, Idrus Z. The Influence of Stable Management and Feeding Practices on the Abnormal Behaviors Among Stabled Horses in Malaysia. J Equine Vet Sci. 2020;94. doi:10.1016/J.JEVS.2020.103230
4. Hanis F, Chung ELT, Kamalludin MH, Idrus Z. Discovering the relationship between dietary nutrients and cortisol and ghrelin hormones in horses exhibiting oral stereotypic behaviors: A review. Journal of Veterinary Behavior. 2020;39: 90–98. doi:10.1016/J.JVEB.2020.05.012
5. Moberg GP, Mench JA. The biology of animal stress : basic principles and implications for animal welfare / ed. by G. P. Moberg, J. A. Mench. 2000 [cited 15 May 2024]. Available:

http://search.ebscohost.com/login.aspx?direct=true&db=edsnuk&AN=edsnuk.vtls001583775&lang=fr&site=eds-live
6. Nagy K, Bodó G, Bárdos G, Bánszky N, Kabai P. Differences in temperament traits between crib-biting and control horses. Appl Anim Behav Sci. 2010;122: 41–47. doi:10.1016/j.applanim.2009.11.005
7. Clegg HA, Buckley P, Friend MA, McGreevy PD. The ethological and physiological characteristics of cribbing and weaving horses. Appl Anim Behav Sci. 2008;109: 68–76. doi:10.1016/J.APPLANIM.2007.02.001
8. Sarrafchi A, Blokhuis HJ. Equine stereotypic behaviors: Causation, occurrence, and prevention. 2013 [cited 15 May 2024]. doi:10.1016/j.jveb.2013.04.068
9. Nicol C. Understanding equine stereotypies. Equine Vet J. 1999;31: 20–25. doi:10.1111/J.2042-3306.1999.TB05151.X
10. Hausberger M, Gautier E, Biquand V, Lunel C, Jégo P. Could Work Be a Source of Behavioural Disorders? A Study in Horses. PLoS One. 2009;4. doi:10.1371/journal.pone.0007625
11. Briefer Freymond S, Ruet A, Grivaz M, Fuentes C, Zuberbühler K, Bachmann I, et al. Stereotypic horses (Equus caballus) are not cognitively impaired. Anim Cogn. 2019;22: 17–33. doi:10.1007/S10071-018-1217-8/FIGURES/3
12. Briefer Freymond S, Beuret S, Ruet A, Zuberbühler K, Bachmann I, Briefer EF. Stereotypic behaviour in horses lowers stress but not spatial learning performance. Appl Anim Behav Sci. 2020;232. doi:10.1016/j.applanim.2020.105099
13. McBride SD, Cuddeford D. The Putative Welfare-Reducing Effects of Preventing Equine Stereotypic Behaviour. Animal Welfare. 2001;10: 173–189. doi:10.1017/S0962728600023848
14. Fureix C, Benhajali H, Henry S, Bruchet A, Prunier A, Ezzaouia M, et al. Plasma cortisol and faecal cortisol metabolites concentrations in stereotypic and non-stereotypic horses: Do stereotypic horses cope better with poor environmental conditions? BMC Vet Res. 2013;9: 1–10. doi:10.1186/1746-6148-9-3/FIGURES/3
15. Sikorska U, Maśko M, Ciesielska A, Zdrojkowski Ł, Domino M. Role of Cortisol in Horse's Welfare and Health. Agriculture 2023, Vol 13, Page 2219. 2023;13: 2219. doi:10.3390/AGRICULTURE13122219
16. Pell SM, McGreevy PD. A study of cortisol and beta-endorphin levels in stereotypic and normal Thoroughbreds. Appl Anim Behav Sci. 1999;64: 81–90. doi:10.1016/S0168-1591(99)00029-5

17. Hemmann K, Raekallio M, Kanerva K, Hänninen L, Pastell M, Palviainen M, et al. Circadian variation in ghrelin and certain stress hormones in crib-biting horses. Vet J. 2012;193: 97–102. doi:10.1016/J.TVJL.2011.09.027
18. Contreras-Aguilar MD, Henry S, Coste C, Tecles F, Escribano D, Cerón JJ, et al. Changes in saliva analytes correlate with horses' behavioural reactions to an acute stressor: A pilot study. Animals. 2019;9. doi:10.3390/ani9110993
19. Nater UM, Rohleder N. Salivary alpha-amylase as a non-invasive biomarker for the sympathetic nervous system: current state of research. Psychoneuroendocrinology. 2009;34: 486–496. doi:10.1016/J.PSYNEUEN.2009.01.014
20. Strahler J, Skoluda N, Kappert MB, Nater UM. Simultaneous measurement of salivary cortisol and alpha-amylase: Application and recommendations. Neurosci Biobehav Rev. 2017;83: 657–677. doi:10.1016/J.NEUBIOREV.2017.08.015
21. Chojnowska S, Ptaszyńska-Sarosiek I, Kępka A, Knaś M, Waszkiewicz N. Salivary Biomarkers of Stress, Anxiety and Depression. Journal of Clinical Medicine 2021, Vol 10, Page 517. 2021;10: 517. doi:10.3390/JCM10030517
22. Sánchez-Amate MC, Flores P, Sánchez-Santed F. Effects of chlorpyrifos in the plus-maze model of anxiety. Behavioural pharmacology. 2001;12: 285–292. doi:10.1097/00008877-200107000-00007
23. Archana E, Pai P, Prabhu BK, Shenoy RP, Prabhu K, Rao A. Altered biochemical parameters in saliva of pediatric attention deficit hyperactivity disorder. Neurochem Res. 2012;37: 330–334. doi:10.1007/S11064-011-0616-X
24. Bazzano M, Laghi L, Zhu C, Lotito E, Sgariglia S, Tesei B, et al. Exercise Induced Changes in Salivary and Serum Metabolome in Trained Standardbred, Assessed by 1H-NMR. Metabolites. 2020;10: 1–14. doi:10.3390/METABO10070298
25. Tuttolomondo D, Antonelli R, Setti G, Ardissino D, Pertinhez T, Gallo M, et al. Salivary biomarkers for diagnosis of acute myocardial infarction: A systematic review. Int J Cardiol. 2023;371: 54–64. doi:10.1016/J.IJCARD.2022.09.043
26. Malamud D, Rodriguez-Chavez IR. Saliva as a Diagnostic Fluid. Dent Clin North Am. 2011;55: 159. doi:10.1016/J.CDEN.2010.08.004
27. Contreras-Aguilar MD, Hevia ML, Escribano D, Lamy E, Tecles F, Cerón JJ. Effect of food contamination and collection material in the measurement of biomarkers in saliva of horses. Res Vet Sci. 2020;129: 90–95. doi:10.1016/J.RVSC.2020.01.006

28. McGreevy P, Nicol C. Physiological and behavioral consequences associated with short-term prevention of crib-biting in horses. Physiol Behav. 1998;65: 15–23. doi:10.1016/S0031-9384(98)00070-5
29. Bachmann I, Bernasconi P, Herrmann R, Weishaupt MA, Stauffacher M. Behavioural and physiological responses to an acute stressor in crib-biting and control horses. Appl Anim Behav Sci. 2003;82: 297–311. doi:10.1016/S0168-1591(03)00086-8
30. Massányi M, Halo M, Mlyneková E, Kováčiková E, Tokárová K, Greń A, et al. The effect of training load stress on salivary cortisol concentrations, health parameters and hematological parameters in horses. Heliyon. 2023;9. doi:10.1016/J.HELIYON.2023.E19037
31. Kułak-Bejda A, Waszkiewicz N, Bejda G, Zalewska A, Maciejczyk M. Diagnostic Value of Salivary Markers in Neuropsychiatric Disorders. Dis Markers. 2019;2019. doi:10.1155/2019/4360612
32. Freymond SB, Bardou D, Beuret S, Bachmann I, Zuberbühler K, Briefer EF. Elevated sensitivity to tactile stimuli in stereotypic horses. Front Vet Sci. 2019;6: 443655. doi:10.3389/FVETS.2019.00162/BIBTEX
33. Briefer Freymond S, Bardou D, Briefer EF, Bruckmaier R, Fouché N, Fleury J, et al. The physiological consequences of crib-biting in horses in response to an ACTH challenge test. Physiol Behav. 2015;151: 121–128. doi:10.1016/J.PHYSBEH.2015.07.015
34. Aurich J, Wulf M, Ille N, Erber R, von Lewinski M, Palme R, et al. Effects of season, age, sex, and housing on salivary cortisol concentrations in horses. Domest Anim Endocrinol. 2015;52: 11–16. doi:10.1016/j.domaniend.2015.01.003
35. Contreras-Aguilar M, Escribano D, Martínez-Subiela S, Martín-Cuervo M, Lamy E, Tecles F, et al. Changes in saliva analytes in equine acute abdominal disease: a sialochemistry approach. BMC Vet Res. 2019;15. doi:10.1186/s12917-019-1933-6
36. Schrödl W, Büchler R, Wendler S, Reinhold P, Muckova P, Reindl J, et al. Acute phase proteins as promising biomarkers: Perspectives and limitations for human and veterinary medicine. Proteomics Clin Appl. 2016;10: 1077–1092. doi:10.1002/PRCA.201600028
37. Kusmawan D, Rini WNE, Aurora WID. Relation of Salivary Alpha-Amylase (sAA) Concentration to Fatigue Biomarkers in Palm Oil Office Workers in Jambi Province: Preliminary Study. Makara J Sci. 2023;27: 289–297. doi:10.7454/mss.v27i4.1450
38. Contreras-Aguilar MD, Cerón JJ, Muñoz A, Ayala I. Changes in saliva biomarkers during a standardized increasing intensity field exercise test in endurance horses. Animal. 2021;15. doi:10.1016/j.animal.2021.100236

39. Bazzano M, Marchegiani A, La Gualana F, Petriti B, Spaterna A, Laus F. Salivary analysis to unveil the paradigma of stress of domestic horses reared in the wild. Sci Rep. 2024;14: 11266. doi:10.1038/s41598-024-62172-2

Funding

This research received no funding

Competing interests

The authors declare no competing interests.
