# Supplementary material for: Competition and stereotypic behavior in Thoroughbred horses: The value of saliva as a diagnostic marker of stress
Source: PLoS One. 2024 Oct 7;19(10):e0311697. doi: 10.1371/journal.pone.0311697 (PMC11458048; doi:10.1371/journal.pone.0311697)
Supplement: Attachment — Submitted filename: Review.pdf [file pone.0311697.s004.pdf]

This paper tries to evaluate the very interesting and necessary objective of eliciting how chronic stress and its negative consequences (in this sense, stereotypic behaviours) can vary some biomarkers (in this case, from saliva) in domestic animals (in this case, in horses) to increase the knowledge in how these states can influence the internal physiological mechanisms, but also to find a possible indicator of welfare status to improve our domestic animal wellbeing. However, how this study is designed and some concerns about how it is described have made this reviewer decides to only accept this study based on the answers to the questions she has asked throughout the manuscript.

Please answer the following questions in detail:

ABSTRACT:

Line 24: “(…); Salivary evaluation (…)”. Please include “salivary biomarkers evaluation” in this sentence.

Line 25: Please replace this sentence with the following: “(…) saliva collection easy to obtain and noninvasive.”

Line 26: Please consider the re-write sentence as advising: “ (…) that salivary cortisol concentrations and alpha-amylase (sAA) and butyrylcholinesterase (BChE) activities, considered as stress biomarkers, may be influenced in horses trained for racing competition with stereotypic behaviours.”.

Line 27: Please include the following information: “Saliva at rest conditions was obtained (…)”

INTRODUCTION:

Line 44: “(…) without a defined function [1].”. Although well referenced, this reviewer also advises the authors to read the book where defining these behaviour conditions performed by Overall et al. 2013 and Mason, 2006. Please include them as references.

- Overall KL. Manual of Clinical Behavioral Medicine for Dogs and Cats. First. Elsevier; 2013. Available: https://books.google.es/books?id=ANzWPAAACAAJ

- Mason G, Rushen J. Stereotipic Aniaml Behaviour. Fundamentals and Applications to Welfare. Second ed. London: CABI; 2006. This reference has also been added as part of this paper’s list of references. However, the year of publication is not correct. Please check it.

Lines 51-53: Please include reference/s.

Line 59: Please specify which drug the authors want to refer to. Some psychotropic drugs, mainly serotonin antagonists and reuptake inhibitors, are used to help in the strategies for solutions to these disorders. Therefore, these drugs could help in dealing

with these stressful situations that they don't know how to resolve. However, it is true that in horses, due to their causes-environment (stress) and not at all by diseases (e.g., neurological diseases), those drugs are not employed and only improving their environment (environment enrichment) can be resolved If they have not already been emancipated. In addition, some of them have not been proven its real security in horses.

Line 66: Please remove “metabolites” and substitute it with “biomarkers”

Line 83: Please change this acronym to “BChE”. Revise it in the document.

Line 84: Alpha amylase and butyrylcholinesterase have been previously defined. Please use the acronym employed previously. In addition, the assays employed by the authors to measure the levels of sAA and BChE do not evaluate concentration but activities. Please correct this.

Line 85: Please modify this sentence as follows: “(…) in three sport horses groups, (…)”.

Line 87: Please modify this sentence as follows: “(…) correlated with stress salivary biomarkers measured at rest conditions (…)”.

METHODS:

Line 100: When describing a group during a paper, the authors must try to use the same definitions for those groups to make it easier for the lecturer to follow the document. Therefore, include in parenthesis the following: “high-level competition horses”. In addition, revise the rest of the document (including the “abstract” section), where groups S and L must be defined as “stereotypic competition horses” and “leisure non-competition horses”, respectively.

Line 100: If the authors used “ten” in the Abstract section, use “ten” here, not the numerical option, or vice versa.

Why did the authors select the number of horses employed in this study? Do the authors know the statistical power of their results? If not, it would be necessary to calculate them.

Lines 99-107: More information about their training and physical conditions are necessary. Which competitions do they usually go to? What Body condition score do they have, and How was it measured? Are they from the same riding school? What are their breeds? What kind of leisure activities were performed in group L? Are there differences in their feeding?

Line 111: Since saliva at rest conditions can be influenced by multiple situations, authors must inform about all the external conditions that could affect the evaluation at rest conditions. In this sense, how were the temperatures and humidities at the sampling collection? Were the samples obtained on the same day or different days?

In addition, Were their mouths previously cleaned? What was the colour of the saliva when it was obtained? It is reported that the presence of feed can interfere with some of the stress biomarkers measured, also correlated with the colour obtained according to the dirtiness degree: Contreras-Aguilar MD, Luisa M, Escribano D, Lamy E, Tecles F, Cerón JJ. Effect of food contamination and collection material in the measurement of biomarkers in saliva of horses. Res Vet Sci. 2020;129: 90–95. doi:10.1016/j.rvsc.2020.01.006. This is key to assuming whether the values are acceptable or not.

Despite this, using only one measurement on each animal to evaluate rest conditions is not representative enough. At least two evaluations must be performed on different days. This condition must be reflected as a limitation of this paper to achieve the planned objective from this paper.

Line 113: This reviewer does not have access to see the figures. Therefore, according to the salivary technique collection, she can not evaluate this aspect and determine if 5 minutes is too much or enough. This reviewer requests the authors to provide these figures.

Line 119: Only from group A or also from group S?

Lines 120-121: After saliva collection, was the cotton introduced into the Salivette tube?

Line 125: How long did it take from when they were stored until they were analyzed? There are some of these analytes sensible to the freeze store:

- Barranco T, Rubio CP, Tvarijonaviciute A, Rubio M, Damia E, Lamy E, et al. Changes of salivary biomarkers under different storage conditions: Effects of temperature and length of storage. Biochem Medica. 2019;29. doi:10.11613/BM.2019.010706.

- Escribano D, Contreras-Aguilar MD., Tvarijonaviciute A, Martínez-Miró S, Martínez-Subiela S, Cerón JJ., et al. Stability of selected enzymes in saliva of pigs under different storage conditions: a pilot study. J Vet Med Sci. 2018;80: 1657–1661. doi:10.1103/PhysRevB.80.104433

Lines 127-128: Please modify it: “Salivary cortisol concentrations and sAA and BChE activities were (…)”.

Lines 128-130: Please write the manufacturer specifications.

This reviewer has some concerns about the assays employed: how did the authors use an ELISA kit for the activity results from BChE and sAA? Have they been previously validated in horse saliva? Although the manufacturer shows validation results, additional validation must be performed and published to guarantee a correct analysis of the assays employed.

Line 138: “(…) salivary butyrylcholinesterase, and cortisol. (…)”. They have been previously defined. In addition, always use the same order when the authors want to write them: e.g., salivary cortisol, sAA and BChE.

Line 139: “ (…) statistically different concentration of (…)”. Please replace it with “change in levels of”.

Line 140 and lines 141-142: Please read the comment performed in line 100.

RESULTS:

Line 151: A word at the beginning of a sentence must not be abbreviated. However, use the correct abbreviations for the rest of salivary biomarkers. Also, always use the same order when defining, describing, and discussing the results from the salivary biomarkers used in this study. Consequently, modify it in the rest of the document.

Line 163: It is not necessary to write the statistical analysis used again. Therefore, only describe as follows (e.g.,): “Significant differences for sAA activities among groups (p=0.04) were observed (figure 2), where sAA activity in group L was significantly lower/higher with respect to groups A (p=0.03) and S (p=0.04), respectively.” Revise and modify this paragraph consequently.

Line 164: Please describe if the levels were lower or higher. In addition, this reviewer does not think it would be necessary for Table 1 if the figure is also presented. The levels of the salivary biomarkers (means and SD and/or reference intervals) can be added to the text when the results have been described.

Lines 171-173 (Figure 2): The figure Legend must describe the figure. Independently, the rest of the document. This means that if a lecturer reads the figure without accessing the rest of the document, they must understand everything. Please modify it consequently. Also, guarantees uniformity in the order of the salivary biomarker, how to describe the groups, etc; with respect to the rest of the document.

DISCUSSION:

Line 184: Please release “metabolites” for “biomarkers”.

Lines 184-185: Well, this answers this reviewer's concern, commented in line 111. However, in this reviewer's experience, although then feeding, the horses always have some of the feed in their mouth since they are almost always eating hay/straw.

Therefore, this review would like to know the dirtiness degree of the samples obtained by these authors according to the scale described in the previously referenced study.

Line 190: Please avoid the first person. Revise the rest of the document and modify it consequently.

Line 207: Please use their abbreviations. (sAA and BChE).

Lines 214-216: This reviewer disagrees with this affirmation. She hypothesizes that this salivary biomarker only evaluates acute stress and not chronic stress.

CONCLUSION (lines 217-243): This cannot be considered as a conclusion from the results obtained in this study. Please modify it according to the results obtained and reflect the objectives described for this paper.
