# Supplementary material for: Competition and stereotypic behavior in Thoroughbred horses: The value of saliva as a diagnostic marker of stress
Source: PLoS One. 2024 Oct 7;19(10):e0311697. doi: 10.1371/journal.pone.0311697 (PMC11458048; doi:10.1371/journal.pone.0311697)
Supplement: Attachment — Submitted filename: Response to reviewers.docx [file pone.0311697.s003.docx]

Dear Reviewers, on behalf of all other Authors, I would thank for the time and efforts spent in reviewing the manuscript and for the points raised, which allow to improve the overall quality of the paper. We have carefully taken into account your suggestions and addressed them throughout thew text, using the track change mode.

In addition, English language has been reviewed by a native speaker.

Below, in bold blue type, the point-to-point reply to reviewers

Reviewer #1: The only real correction that must be made is to changes agonistic to competition (or show). Agonistic means aggressive in English). In the discussion a comparison should be made to the findings of Hausberger. **Text has been implemented to reflect this comment**

46 Stereotypies are not communicative; they do not send messages to others **Text has been changed to reflect this comment**

53 stereotypic behavior **amended**

55 stereotypies **amended**

67 pointed to **amended**

69 In addition to cortisol **amended**

because it is noninvasive

97 for at least the prior year **amended**

120 supernatants **amended**

133 afterwards

174 of accurately assessing **amended**

178 and so avoided that contamination **amended**

186 were these horses racing or were they competing in cross country or stadium jumping. **amended**

216 free ranging not wild **amended**

219 may have caused **amended**

239stereotypic **amended**

Reviewer #2: This submission has the potential to provide helpful information to the horse behavior research community. However, in its current format, it is difficult to fully comprehend the message. Please work on the English grammar & sentence structure to help with clarity. (I have only thoroughly addressed the comments on the abstract.)

Please review the attached document. **The document has been partially rewritten and checked by a native English speaker to ensure clarity of the text. We have carefully addressed all the comments highlighted in the attached file of the email we received directly in the manuscript text**
