# Supplementary material for: Competition and stereotypic behavior in Thoroughbred horses: The value of saliva as a diagnostic marker of stress
Source: PLoS One. 2024 Oct 7;19(10):e0311697. doi: 10.1371/journal.pone.0311697 (PMC11458048; doi:10.1371/journal.pone.0311697)
Supplement: Attachment — Submitted filename: Response to reviewer 3.docx [file pone.0311697.s006.docx]

Dear Reviewer, on behalf of all other Authors, I would thank for the time and efforts spent in reviewing the manuscript and for the points raised, which allow to improve the overall quality of the paper. We have carefully taken into account your suggestions and addressed them throughout thew text, using the track change mode.

Below, in bold blue type, the point-to-point reply to reviewers

Reviewer #3: Abstract:
- Line 26-27: rewrite to: In this study, we hypothesized that salivary cortisol concentrations, in addition to alpha-amylase (sAA) and butyrylcholinesterase (BChE) activities are considered …….etc **Text modified to reflect this comment**
- Line 28: write (that) before (may) **Text modified to reflect this comment**
Material and methods
- Line 145-147: revise. **Text revised for clarity**
Results
- N.B. write subheadings
- Line 172-173: add to methods line 145
- Line 174-190: too long, better presented in table and only mention important information in text.

- Cortisol in figure 2 missing the comparison between S and L, S and A.

**Result section has been modified accordingly to your comments.**
